# Supplementary material for: Hydrothermal Cobalt Doping of Titanium Dioxide Nanotubes towards Photoanode Activity Enhancement
Source: Materials (Basel). 2021 Mar 19;14(6):1507. doi: 10.3390/ma14061507 (PMC8003354; doi:10.3390/ma14061507)
Supplement: Supplementary file 1 [file materials-14-01507-s001.pdf]

materials

Supplementary

Hydrothermal Cobalt Doping of Titanium Dioxide Nanotubes towards Photoanode Activity Enhancement

Mariusz Wtulich [1], Mariusz Szkoda [1], Grzegorz Gajowiec [2], Maria Gazda [3], Kacper Jurak [4], Mirosław Sawczak [5] and Anna Lisowska-Oleksiak [1,*]

[1] Department of Chemistry and Technology of Functional Materials, Chemical Faculty, Gdańsk University of Technology, Gdańsk 80-233, Poland; wturglish@gmail.com (M.W.); mariusz.szkoda1@pg.edu.pl (M.S.)
[2] Institute of Machine Technology and Materials, Faculty of Mechanical Engineering and Ship Technology, Gdansk University of Technology, Gdansk 80-233, Poland; grzgajow@pg.edu.pl
[3] Department of Solid State Physics, Faculty of Applied Physics and Mathematics, Gdansk University of Technology, Gdansk 80-233, Poland; maria.gazda@pg.edu.pl
[4] Department of Electrochemistry, Corrosion and Materials Engineering, Chemical Faculty, Gdansk University of Technology, Gdańsk 80-233, Poland; kacper.jurak@pg.edu.pl
[5] The Szewalski Institute of Fluid-Flow Machinery, Polish Academy of Sciences, Gdańsk 80-231, Poland; mireks@imp.gda.pl
* Correspondence: alo@pg.edu.pl

Citation: Wtulich, M.; Szkoda, M.; Gajowiec, G.; Gazda, M.; Jurak, K.; Sawczak, M.; Lisowska-Oleksiak, A. Hydrothermal Cobalt Doping of Titanium Dioxide Nanotubes towards Photoanode Activity Enhancement. *Materials* **2021**, *14*, 1507. https://doi.org/10.3390/ma14061507

Academic Editor: Rosalinda Inguanta

Received: 18 February 2021
Accepted: 15 March 2021
Published: 19 March 2021

Publisher's Note: MDPI stays neutral with regard to jurisdictional claims in published maps and institutional affiliations.

Copyright: © 2021 by the authors. Licensee MDPI, Basel, Switzerland. This article is an open access article distributed under the terms and conditions of the Creative Commons Attribution (CC BY) license (http://creativecommons.org/licenses/by/4.0/).

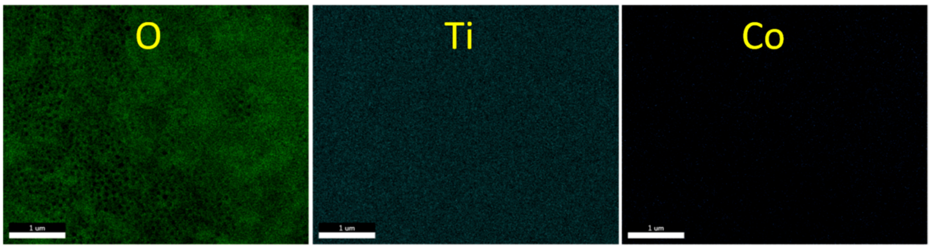

Figure S1. EDX maps of the O, Ti and Co distributions.
